# Antimicrobial Resistance, SCC*mec*, Virulence and Genotypes of MRSA in Southern China for 7 Years: Filling the Gap of Molecular Epidemiology

**DOI:** 10.3390/antibiotics12020368

**Published:** 2023-02-10

**Authors:** Junyan Liu, Tengyi Huang, Thanapop Soteyome, Jian Miao, Guangchao Yu, Dingqiang Chen, Congxiu Ye, Ling Yang, Zhenbo Xu

**Affiliations:** 1 College of Light Industry and Food Science, Guangdong Provincial Key Laboratory of Lingnan Specialty Food Science and Technology, Academy of Contemporary Agricultural Engineering Innovations, Zhongkai University of Agriculture and Engineering, Guangzhou 510225, China; 2 Key Laboratory of Green Processing and Intelligent Manufacturing of Lingnan Specialty Food, Ministry of Agriculture, Guangzhou 510225, China; 3 Department of Laboratory Medicine, The Second Affiliated Hospital of Shantou University Medical College, Shantou 515063, China; 4 Home Economics Technology, Rajamangala University of Technology Phra Nakhon, Bangkok 10300, Thailand; 5 Graduate Program in Pharmaceutical Sciences, College of Graduate Health Sciences, University of Tennessee Health Science Center, Memphis, TN 38163, USA; 6 Center of Clinical Laboratory Medicine, First Affiliated Hospital of Jinan University, Guangzhou 510620, China; 7 Department of Laboratory Medicine, Zhujiang Hospital, Southern Medical University, Guangzhou 510282, China; 8 Department of Dermato-Venereology, Third Affiliated Hospital of Sun Yan-Sen University, Guangzhou 510630, China; 9 Department of Laboratory Medicine, The First Affiliated Hospital of Guangzhou Medical University, Guangzhou Medical University, Guangzhou 510120, China

**Keywords:** MRSA, SCC*mec*, virulence genes, MLST, *spa* types

## Abstract

As the prevalence of *Staphylococcus aureus* infections is of worldwide concern, phenotype and genotype in prevalent MRSA strains require longitudinal investigation. In this study, the antibiotic resistance, virulence gene acquisition, and molecular type were determined on a large scale of nosocomial *S. aureus* strains in Southern China during 2009–2015. Bacterial identification and antimicrobial susceptibility to 10 antibiotics were tested by Vitek-2. Virulence genes encoding staphylococcal enterotoxins (SEA, SEB, SEC, SED, and SEE), exfoliative toxins (ETA and ETB), Panton–Valentine leukocidin (PVL), and toxic shock syndrome toxin (TSST) were detected by PCR, with SCC*mec* typing also conducted by multiplex PCR strategy. Genotypes were discriminated by MLST and spaA typing. MLST was performed by amplification of the internal region of seven housekeeping genes. PCR amplification targeting the *spa* gene was performed for spa typing. No resistance to vancomycin, linezolid, or quinupristin and increase in the resistance to trimethoprim/sulfamethoxazole (55.5%) were identified. A total of nine SCC*mec* types and subtypes, thirteen STs clustered into thirteen *spa* types were identified, with ST239-SCC*mec* III-t037 presenting the predominant methicillin-resistant *S. aureus* (MRSA) clone. Typically, SCC*mec* type IX and ST546 were emergent types in China. Isolates positive for both *pvl* and *tsst* genes and for both *eta* and *etb* genes were also identified. Important findings in this study include: firstly, we have provided comprehensive knowledge on the molecular epidemiology of MRSA in Southern China which fills the gap since 2006 or 2010 from previous studies. Secondly, we have presented the correlation between virulence factors (four major groups) and genotypes (SCC*mec*, ST and *spa* types). Thirdly, we have shown evidence for earliest emergence of type I SCC*mec* from 2012, type VI from 2009 and type XI from 2012 in MRSA from Southern China.

## 1. Introduction

As a major human pathogen and important “superbug”, methicillin-resistant *Staphylococcus aureus* (MRSA) is responsible for a wide range of infections and diseases due to possession of numerous virulence factors and toxins [1,2,3,4]. Currently, indiscriminate use of existing antibiotics which lead to the spread of antibiotic resistance, poses a dilemma for the treatment of bacterial infections [5,6,7,8,9]. Despite a variety of novel therapy and drug discovery, antibiotic resistance in microbes remains a major public health concern for the treatment of infectious diseases [10,11,12,13,14]. MRSA is one of such infectious bacteria that has become resistant to β-lactam antibiotics [15,16,17,18]. As China remains one of the worst areas for antibiotic abuse [19], it is necessary to raise general concerns regarding the surveillance and investigation of antibiotic resistance mechanism involved in clinical MRSA in China. To better understand the molecular evolution and epidemiological characterization of *S. aureus*, the analysis of SCC*mec* and genetic backgrounds mainly involved in randomly amplified polymorphic DNA (RAPD) types, sequence types (STs), clone complexes (CCs), and *spa* types is necessary [20,21,22].

In China, the first evidence of molecular types in MRSA dates back to 2003. However, this study had been primarily conducted using a large number of samples (118) from a hospital in Taiwan with a much smaller number of samples (14) from a hospital in Nanjing in mainland China [23]. In 2007, we reported on ST239-MRSA and strains carrying class 1 integrons isolated in 2005. Ref. [5], for which we had further provided the SCC*mec* (type III), *spa,* and *coa* types in a subsequent study [24]. In 2008, we reported on the presence of the SCC*mec* of four different types of methicillin-resistant coagulase-negative staphylococci (MRCNS) [25]. In 2011, we conducted a molecular epidemiology study on MRSA isolates from 2001–2006, including SCC*mec*, RAPD, MLST, *spa,* and *coa* typing, as well as carriage of class 1 integrons [26]. In a subsequent study, we had provided evidence on the molecular epidemiology of MRSA during 2001–2010 [27]. As found from all above studies conducted in Guangzhou representative of Southern China, ST239-MRSA-III was highly prevalent, with only a few exceptions carrying type II SCC*mec* and none of other ST than ST239. Only one distinct *coa* type HIJKL and 2 *spa*A types (WGKAOMQ-t037 and WGKAQQ-t030 were identified during the study period of 2001–2010.

However, since the types of SCC*mec* has increased from six types (I to VI) in 2006 to fourteen (I to XIV) to date, the concern about the evolution of SCC*mec* and molecular epidemiology in MRSA since 2006 or 2010 in Southern China remains unclear but important [28,29]. Consequently, in this study, numerous *S. aureus* strains isolated in Southern China during 2009–2015, were subjected to antimicrobial resistance, virulence genes identification, SCC*mec*, MLST, and *spa* typing.

## 2. Results

### 2.1. Antimicrobial Susceptibility Profile

For 524 *S. aureus* strains isolated from various departments, infection sites, and patients covering different age groups (Table 1), 490 (93.5%) MRSA and 34 methicillin-susceptible *S. aureus* (MSSA) were identified, respectively (Table 2). No resistance to vancomycin, linezolid, or quinupristin was detected, yet a percentage of the strains investigated demonstrated resistance to oxacillin (93.9%), erythromycin (83.9%), ciprofloxacin (80.0%), levofloxacin (79.5%), moxifloxacin (73.4%), clindamycin (73.1%), trimethoprim/sulfamethoxazole (55.5%) and rifampin (34.7%), totaling the multi-drug resistance rate as 88.7% (465/524).

### 2.2. Carriage of Virulence Genes

According to the results, SEs genes were commonly detected, with the identification rate of *sea*, *seb*, *sec*, *sed,* and *see* found to be 67.8% (352/519), 33.0% (171/519), 42.6% (221/519), 1.9% (10/519), 35.8% (186/519), respectively. For exfoliative toxins genes, 12.4% (61/493) and 9.6% (49/511) of the strains were positive for *eta* and *etb*. In addition, *tsst* and *pvl* were detected in 28.6% (146/511) and 14.9% (76/511) of strains.

### 2.3. SCCmec Types

As shown by the results of SCC*mec* typing, a total of 7 types and 2 subtypes were identified within 490 MRSA isolates (Table 2), including types I/IA, II, III/IIIA, IV, V, VI, and IX. The most prevalent SCC*mec* was type III (48.0%, 235/490, with 9.2% for subtype IIIA), followed by type II (18.6%, 91/490), IV (13.9%, 68/490), VI (6.3%, 31/490), V (3.7%, 18/490) and I (3.3%, 16/490, with 3.3% for subtype IA).

### 2.4. MLST

Clonal relatedness of 508 *S. aureus* strains was investigated by DNA fingerprinting by RAPD-PCR. A total of 112 distinctive RAPD types were identified, 71 RAPD types were at least represented by 2 strains, 41 RAPD types were only represented by one strain. The 71 RAPD types with at least 2 strains were further clustered into 13 STs by MLST (Table 3). The occurrence of ST239, ST5, ST45, ST59, and ST1 was found to be 65.5% (306/467), 9.0% (42/467), 7.5% (35/467), 5.4% (25/467), and 2.1% (10/467).

### 2.5. Spa Type

A total of 13 *spa* types were identified by *spa* typing (Table 3). Amongst these, t037 was the most prevalent type (38.5%, 180/467), followed by t030 (26.6%, 124/467), t1081 (14.6%, 68/467), t437 (5.8%, 27/467), and t002 (5.8%, 27/467).

## 3. Discussion

A remarkably high multi-drug resistance rate (88.7%) was obtained in the tested strains, with the majority as MRSA. Noteworthily, increase in trimethoprim/sulfamethoxazole resistance (from 20.3% during 2009–2012 to 82.7% during 2013–2015, particularly 100% in 2014 and 2015) and a decrease in ciprofloxacin resistance (from 92.0% in 2009 to 70.8% in 2015) were obtained (Table 2). An increase in trimethoprim/sulfamethoxazole resistance within *S. aureus* isolates had also been reported in our previous study and other reports [30,31,32,33]. A decrease in ciprofloxacin in this study was also in accordance with the previous surveillance, whereas the resistance rate of ciprofloxacin was found to be 69.2% during 2006–2010 and 44.0% in 2011–2015. Concerning the correlation between antibiotic resistance and SCC*mec* types, isolate type II SCC*mec* showed significantly lower resistance to erythromycin (8.3%) than other types (average 81.5%). Type V SCCmec (23.5%) was the least resistant to trimethoprim/sulfamethoxazole, followed by type II (42.9%) and type III (44.4%). Low resistance to rifampin was also identified in type II SCC*mec* (18.3%)

As a major human pathogen, MRSA has a number of virulence genes in its genome, which contributes to its pathogenicity. However, very few studies had touched upon the relevance between its molecular epidemiology and virulome (virulence genes profile). In *S. aureus*, extracellular protein toxins are major toxins that could significantly enhance its pathogenicity, including Staphylococcal enterotoxins (SEs), toxic shock syndrome toxin 1 (TSST-1), exfoliative toxins (ETs) and Panton–Valentine leukocidin (PVL) [34,35]. In addition, there are a few subtypes of such toxins, especially SEs. In this study, four types of such toxins with a total of nine targets were investigated, followed by a comprehensive analysis of the relatedness. For the occurrence of virulence genes, high diversity was found in different years and subtypes. For SEs, *sea*, *seb,* and *sec* had been commonly detected during 2009–2015, with *sea* being the most prevalent type. In comparison, the identification rate of *see* had decreased in 2011 and 2013, but with the highest rate in 2012. While *sed* had only been detected in 2011 and 2012. Change in the detection of *sec* (rise from 8.0% in 2009 to 62.8% in 2015) and *see* (drop from 73.0% in 2009 to 13.2% in 2015) was also observed (Table 2). The other four virulence genes were also commonly detected during the studied period. Importantly, first emerging in 2010, the carriage of *eta* changed from 7.0% during 2009–2012 to 29.4% in 2015 [36]. In this study, we further performed a comprehensive analysis of the relatedness between virulence genes and other microbial traits, including antibiotic susceptibility, SCC*mec*, MLST, and *spa* types. However, no significant correlation was identified between virulence gene carriage and antibiotic susceptibility. Table 4 had comprehensively shown the relatedness between the carriage of each specific virulence gene, with SCC*mec*, ST types, and *spa* types separately. For SEs, a higher relatedness rate with type I SCC*mec* was found, compared with a much lower rate with hospital-associated MRSA (type II and III SCC*mec*), especially for seb and sed. In addition, it was found that sea showed a higher prevalence in ST239 than other SEs. For *tsst* and *pvl*, the higher occurrence was found in CA-MRSA.

Previously in Southern China, type III SCC*mec* was the dominant with only a few strains carrying type II SCC*mec* or untypeable [26,27]. However, in this study, diversity in SCC*mec* was obtained, with type I/IA, II, III/IIIA, IV, V, VI and IX SCC*mec* totaling 7 types and 2 subtypes within 490 MRSA isolates (Table 2). Despite the high occurrence of type III SCC*mec*, declination in hospital associated types (70.4% with 345/490 for I, II and III), increase in community-associated types (17.6% with 86/490 for IV and V), as well as diversity in detected SCC*mec*, had been shown in the present study. Remarkably, the five types (I, IV, V, VI and IX) and one subtype (IA) newly found in this study, had represented the first evidence of type I (mainly in Japan and Korea) and IX (the second identification after Thailand) in China. In addition, prevalence of type III had dropped from higher than 90% to 48.0%, with an increase in type II to 18.6%. Further analysis was conducted in regards with the correlation between virulence factor and SCC*mec*. Frequently identified in community-associated MRSA (CA-MRSA) strains [37], *pvl* was detected in only 14.9% of tested strains, of which type II (35.5%) and III (34.2% including 7.9% IIIA) SCC*mec* were predominant followed by type IV (19.7%), VI (3.9%) and I (2.6%). Remarkably, 24 and 12 *S. aureus* isolates were *pvl*^+^*tsst*^+^ and *eta*^+^*etb*^+^, respectively.

As genotyping was concerned, the strategy we used was to combine MLST and *spa* typing. In comparison with previous reports, higher diversity was found in this study, with 7 types and 2 subtypes of SCC*mec*, 13 STs, and 13 *spa* types identified from 490 MRSA strains within a study period of 7 years and 2 medical centers. Within STs and *spa* types, despite ST239 remaining as the dominant type, common SCC*mec* types such as ST5, ST45, and ST59 had also been frequently detected. Significantly, ST546 (2.1%, 10/467) has been first identified in Asia during 2010–2012, and the carriage rate of ST239 dropped from 76.9% in 2011 to 58.5% in 2015. For clonal complex (CC) as shown in Table 4, ST239-SCC*mec* II/III/IV, ST5/45-SCC*mec* III, ST59-SCC*mec* II, ST546-SCC*mec* II/III/IV were the prevalent types. In Southern China, ST239-SCC*mec* III was the only predominant MRSA clone during 2001–2006 (93.8%) and had changed to 37.7% during 2009–2015. Diversity of both SCC*mec* and ST were also found, with ST239-SCC*mec* IV, ST5-SCC*mec* III, ST45-SCC*mec* III, ST59-SCC*mec* II, ST1-SCC*mec* III, ST546-SCC*mec* II, III and ST546-SCC*mec* IV identified for the first time. In combination with SCC*mec* ST and *spa* types, ST239-SCC*mec* II/III-t037/t030/t1080 was the predominant MRSA clone accounting for 30% of all tested MRSA strains (Table 3).

## 4. Materials and Methods

### 4.1. Clinical Samples and Bacterial Strains

From 2009 to 2015, a total of 524 *S. aureus* isolates collected from First Affiliated Hospital of Guangzhou Medical University (FAHGMU) and First Affiliated Hospital of Jinan University (FAHJU) were evaluated. FAHGMU is a grade A tertiary hospital combining medical, teaching, scientific research, health care, rehabilitation, and pre-hospital emergency. It is one of the first 13 national clinical medical research centers in China. FAHJU is a tertiary-level teaching hospital with a large-scale patient population and two 2000-bed medical centers in Southern China. With the advantage of location (in the central city of Southern China) and medical capacity, patients in different areas in Southern China have therapy and treatment in FAHGMU and FAHJU. Thus, the data collected in the current study present the epidemiology of *S. aureus* during the contemporary period in the whole Southern China area. *S. aureus* isolates were maintained as glycerol stock stored at −80 °C. A small amount of *S. aureus* stock was spread onto tryptic soy agar (TSA) and incubated at 37 °C for 24 h to obtain single colonies. A single colony of *S. aureus* was transferred to 2 mL of tryptic soy broth (TSB) and incubated at 37 °C with shaking at 200 rpm overnight prior to further experiments.

### 4.2. Bacterial Identification and Antimicrobial Susceptibility Testing (AST)

For the clinical sample, blood agar plate (Huankai Biotech, Guangzhou, China) was used to specifically identify *S. aureus* strains. After acquisition of yellow/golden yellow colonies, bacterial identification on all *S. aureus* strains was performed to the species level according to standard procedures [38], including colony morphology, Gram staining, catalase test, Vitek-2 automated system, and PCR amplification on *16S rRNA* (*Staphylococcus* specific) and *femA* (*S. aureus* specific) genes. Methicillin resistance was determined by PCR on *mecA* using primers M1 and M2. *S. aureus* strain ATCC29212 carrying *16S rRNA*, *mecA,* and *femA* genes served as positive control. AST was conducted by Vitek-2 [39] with 10 studied antibiotics including ciprofloxacin, clindamycin, erythromycin, levofloxacin, linezolid, moxifloxacin, oxacillin, quinupristin, rifampin, trimethoprim/sulfamethoxazole, and vancomycin. All results were interpreted according to criteria of Clinical and Laboratory Standards Institute (CLSI 2021). Strain resistance to >2 drugs was considered to be multi-drug resistance [40].

### 4.3. Detection of Virulence Genes

A total of 9 virulence genes were selected to test in this study (Table 2), including 5 SEs genes (*sea*, *seb*, *sec*, *sed* and *see*), 2 exfoliative toxins genes (*eta* and *etb*), 1 panton-valentine leukocidin gene (*pvl*) and 1 toxic shock syndrome toxin gene (*tsst*). Genomic DNA from *S. aureus* strains for PCR amplification was prepared from overnight cultures according to the instruction of DNA extraction kit (Dongsheng Biotech Co., Ltd., Guangzhou, China). Briefly, harvested cells were orderly treated with lysozyme and proteinase K to obtain lysates. Suspension was purified after the removal of proteins and salts etc. The highly purified DNA was strictly stored at −20 °C. A total of 9 virulence genes were detected by PCR in all *S. aureus* strains, with tested genes encoding staphylococcal enterotoxins (SEA, SEB, SEC, SED and SEE), exfoliative toxins (ETA and ETB), Panton-Valentine leukocidin (*PVL*) and toxic shock syndrome toxin (*TSST*) [36,41]. All PCR assays were performed in triplicate using the primers listed in Table 5 as described previously [42,43,44]. PCR amplification was performed in a volume of 50 µL with 2× PCR Master Mix (Dongsheng Biotech Co., Ltd., Guangzhou, China). The DNA Thermal Cycler EDC-810 (Eastwin Biotech Co., Ltd., Beijing, China) was programmed as follows: the first denaturation at 94 °C for 5 min, denaturation at 94 °C for 30 s, annealing at respective temperature listed in Table 5 for 30 s, and an extension at 72 °C for 90 s for 30 cycles and at last the final extension at 72 °C for 7 min. Three positive strains for each virulence gene were adapted to Sanger sequencing to confirm the accuracy of the amplicons. Strains with correct amplicons were subsequently used as positive controls.

### 4.4. SCCmec Typing

A multiplex PCR strategy is the most available method for SCC*mec* typing, and it can be used to study the evolution of MRSA [45]. Different SCC*mec* types were determined by specific primers listed in Table 5. PCR amplification was performed in a volume of 50 µL with 2× PCR Master Mix (Dongsheng Biotech Co., Ltd., Guangzhou, China). The DNA Thermal Cycler EDC-810 (Eastwin Biotech Co., Ltd., Beijing, China) was programmed as follows: the first denaturation at 94 °C for 5 min, denaturation at 94 °C for 30 s, annealing at respective temperature listed in Table 5 for 30 s, and an extension at 72 °C for 90 s for 30 cycles and at last the final extension at 72 °C for 7 min. PCR products were analyzed by electrophoresis on 1.5% agarose gel. *S. aureus* strains 10442 (carrying *ccr1* and *IS1272*-*mecA*), N315 (carrying *ccr2* and *mecI*-*mecR1*), JP25 (carrying *ccr3*), and WIS (carrying *IS431*-*mecI*-*mecA*) were served as positive controls.

### 4.5. DNA Fingerprinting Analysis by RAPD-PCR

Random primers AP1, AP7 and ERIC2 (Table 5) [46,47] were applied for RAPD-PCR assay [48]. PCR amplification was performed in a volume of 50 µL with 2× Taq PCR Master Mix (Dongsheng Biotech Co., Ltd., Guangzhou, China). Components for RAPD-PCR including 2× Taq PCR Master Mix 25 µL, primer 3 µL, DNA template 1 µL and ddH_2_O 21 µL. RAPD-PCR program was as follows: 94 °C for 5 min; followed by 94 °C 1 min, 38 °C 1 min, 72 °C 2 min for 8 cycles, and 94 °C 1 min, 38 °C 1 min, 72 °C 2 min for 25 cycles; 72 °C 7 min. PCR products were analyzed by electrophoresis on 1.5% agarose gel.

### 4.6. MLST

Strains adapted for MLST and *spa* typing [20,49,50,51]. MLST was performed by amplification of the internal region of seven housekeeping genes, including *arc*, *aroE*, *glpF*, *gmk*, *pta*, *tpi,* and *yqiL*. The amplicons were 456, 456, 465, 417, 474, 402, and 516 bp, respectively. The PCR products were sequenced with ABI Prism 377 DNA Sequencer (PE Applied Biosystems, USA) and compared with the existing sequences available in the MLST website (http://www.pubmlst.org, accessed on 1 November 2022) for *S. aureus*, and the allelic number was determined for each sequence. The sequence type (ST) was determined according to the pattern of the combination of the seven alleles, and the clonal complex (CC) was defined by the BURST (based upon related sequence types) program v3.0 by accessing the MLST website.

### 4.7. Spa Typing

Protein A, which is encoded by *spa* gene, is a surface protein originally found in the cell wall of *S. aureus* and it has been used in biochemical research because of its ability to bind immunoglobulins. According to the number, characteristics, and arrangement of repeated sequences in X region, which is a highly repeated sequence in protein A, the *spa* typing can reliably and accurately present the polymorphism of *S. aureus* [52]. PCR amplification targeting *spa* gene with primers *spa*-Up and *spa*-Dn (Table 5) was performed for *spa* typing. The PCR was programmed as follows: the first denaturation at 94 °C for 5 min, denaturation at 94 °C for 30 s, annealing at 60 °C for 45 s, and an extension at 72 °C for 90 s for 30 cycles, and at last the final extension at 72 °C for 10 min. PCR products were analyzed by electrophoresis on 1.5% agarose gel. Purified amplicons were sequenced with ABI Prism 377 DNA Sequencer (PE Applied Biosystems, Foster City, CA, USA) and repetitive sequences were collected to align to Ridom *spa*-server (https://spaserver.ridom.de/) (accessed on 4 October 2017) in which 16150 *spa* types, 709 repeats, and 350761 strains are available.

### 4.8. Statistical Analysis

In this study, antimicrobial susceptibility results and organization were managed in WHONET (version 5.6) (Boston, MA, USA). The chi-square test or Fisher’s exact test was applied in the correlation analyses, if appropriate. A *p* value < 0.05 was defined as statistically significant.

## 5. Conclusions

In conclusion, this study has provided comprehensive knowledge on the correlation among antimicrobial resistance, SCC*mec*, ST, and *spa* types from a large scale of clinical *S. aureus* during a lengthy period. An increase in the resistance to trimethoprim/sulfamethoxazole was identified. A total of nine SCC*mec* types and subtypes, thirteen STs clustered into thirteen *spa* types were identified, with ST239-SCC*mec* III-t037 presenting the predominant MRSA clone. Typically, SCC*mec* type IX and ST546 were emergent types in China. Isolates positive for both *pvl* and *tsst* genes and for both *eta* and *etb* genes were also identified. The results yielded from this study will aid in further surveillance of molecular epidemiology and evolutionary discrepancy on MRSA.

## Figures and Tables

**Table 1:** Distribution of *S. aureus* isolated from different clinical samples.

	Isolation Source	Strain Amount	Percentage *
Department	Internal medicine	158	30.15%
	ICU	42	8.02%
	Orthopedic	52	9.92%
	urology	47	8.97%
	Neurology	37	7.06%
	Surgery	32	6.11%
	Pediatrics	16	3.05%
	Obstetrics and Gynecology	4	0.76%
	Other	136	25.95%
Infection site	Sputum	269	51.34%
	Pus	39	7.44%
	Urinary tract	37	7.06%
	Bloodstream	29	5.53%
	Wound	27	5.15%
	Respiratory tract	10	1.91%
	Other	113	21.56%
Age	The old	272	51.91%
	The young and the middle-aged	217	41.41%
	Infant	35	6.68%

* The percentage was calculated by the amount of strains in each isolation source divided by the total amount of strains. There is overlap among isolation sources in the department, infection site, and age.

**Table 2:** Antimicrobial susceptibility, carriage of virulence genes, and SCC*mec* types of *S. aureus* isolates.

		2009 (n = 25)	2010 (n = 23)	2011 (n = 104)	2012 (n = 115)	2013 (n = 81)	2014 (n = 121)	2015 (n = 51)	Total
SCC*mec* *	I	0	0	0	1	3	1	7	12
	IA	0	0	0	1	3	1	2	7
	II	1 ^a^	2 ^ab^	18 ^a^	24 ^a^	16 ^a^	21 ^a^	9 ^a^	91
	III	18 ^d^	12 ^f^	54 ^a^	53 ^b^	16 ^e^	29 ^c^	8 ^f^	190
	IIIA	1 ^b^	0 ^b^	9 ^a^	6 ^b^	7 ^ab^	15 ^a^	7 ^a^	45
	IV	2 ^b^	4 ^b^	8 ^b^	12 ^b^	15 ^a^	20 ^a^	7 ^b^	68
	V	0 ^b^	0 ^b^	1 ^b^	9 ^a^	4 ^ab^	3 ^ab^	1 ^ab^	18
	VI	1 ^b^	0 ^b^	4 ^b^	1 ^b^	7 ^a^	12 ^a^	6 ^a^	31
	Others	0	0	0	1	6	14	3	24
Toxins *	*sea*	21 ^b^	22 ^b^	73 ^a^	71 ^a^	56 ^a^	79 ^a^	30 ^a^	352
	*seb*	9 ^d^	3 ^e^	28 ^c^	32 ^c^	37 ^b^	39 ^a^	23 ^d^	171
	*sec*	2 ^b^	5 ^b^	22 ^b^	52 ^a^	47 ^a^	62 ^a^	32 ^a^	222
	*sed*	0 ^b^	0 ^b^	2 ^b^	8 ^a^	0 ^b^	0 ^b^	0 ^b^	10
	*see*	14 ^d^	17 ^b^	53 ^a^	49 ^a^	2 ^e^	16 ^c^	11 ^e^	186
	eta	0 ^c^	1 ^c^	8 ^c^	9 ^c^	15 ^b^	31 ^a^	15 ^b^	79
	etb	3 ^c^	9 ^b^	16 ^a^	13 ^a^	1 ^c^	6 ^c^	1 ^c^	49
	*pvl*	2 ^d^	13 ^b^	25 ^a^	21 ^a^	3 ^d^	2 ^d^	10 ^c^	76
	*tsst*	0 ^e^	7 ^d^	8 ^d^	17 ^d^	43 ^a^	41 ^b^	30 ^c^	146
Antibiotic resistance #	oxacillin	10 ^b^	19 ^b^	96 ^a^	103 ^a^	68 ^b^	93 ^b^	45 ^b^	434
	trimethoprim/ sulfamethoxazole	3 ^c^	11 ^c^	8 ^c^	14 ^c^	34 ^b^	109 ^a^	48 ^b^	227
	erythromycin	23 ^a^	23 ^a^	90 ^a^	90 ^a^	49 ^a^	87 ^a^	39 ^a^	401
	ciprofloxacin	23 ^c^	21 ^c^	87 ^a^	91 ^a^	54 ^b^	78 ^b^	34 ^b^	388
	rifampin	14 ^c^	9 ^d^	27 ^b^	30 ^b^	40 ^a^	33 ^b^	12 ^d^	165
	moxifloxacin	23 ^b^	15 ^c^	71 ^a^	81 ^a^	53 ^a^	76 ^a^	34 ^a^	353
	penicillin	20 ^c^	21 ^c^	78 ^a^	69 ^a^	46 ^b^	64 ^a^	30 ^b^	328
	tetracycline	17 ^d^	12 ^d^	81 ^a^	37 ^b^	36 ^c^	68 ^b^	35 ^d^	286
	levofloxacin	6 ^b^	12 ^b^	87 ^a^	91 ^a^	53 ^b^	78 ^b^	34 ^b^	361
	clindamycin	17 ^e^	23 ^d^	86 ^a^	71 ^b^	43 ^c^	66 ^c^	29 ^c^	335

* The amount of *S. aureus* isolates identified to be positive for each SCC*mec* types (I, IA, II, III, IIIA, IV, V, VI, and Others) and virulence factors (*sea*, *seb*, *sec*, *sed*, *see*, *eta*, *etb*, *pvl*, and *tsst*) in each year were listed, with total amount of isolates in the rightmost column. # The amount of *S. aureus* isolates resistance to each antibiotic (oxacillin, trimethoprim/sulfamethoxazole, erythromycin, ciprofloxacin, rifampin, moxifloxacin, penicillin, tetracycline, levofloxacin, and clindamycin) in each year were listed. The data were adapted to chi-square test between years and the superscripted “abcde” in the table is the significant difference letter marking method. (Arrange all the averages from large to small, label the largest average with the letter “a”, subtract that average from the following to obtain the range, and label any range less than 0.05 with the letter “a”, until the range is greater than or equal to 0.05, or a significant difference from an average, the average is superscript with the letter “b”, and the average labeled “b” is used as the criterion, compared with the above average which is larger than it, any difference which is not significant is marked with the letter “b”, and the maximum average marked with “b” is used as the standard, compared with the unmarked average below, those that are not significantly different continue to be labeled with the letter “b” until an average that is significantly different is labeled with the letter “c”. The comparisons are repeated until the smallest average has a marked letter).

**Table 3:** Genotypes of *S. aureus* isolated.

Genotypes	2009	2010	2011	2012	2013	2014	2015	Total
ST239-t037	11	6	60	42	9	10	3	141
ST239-t030	5	3	16	13	15	26	15	93
ST239-t1081	4	2	4	11	2	2	0	25
ST59-t437	1	0	4	6	2	2	0	15
ST5-t002	0	0	5	2	0	2	0	9
ST546-t1081	0	4	3	3	0	0	0	10
ST45-t1081	1	0	5	2	0	0	0	8
ST1-t4084	0	0	0	0	1	7	0	8
ST5-t030	0	1	1	5	0	0	0	7
ST5-t1084	0	0	0	0	0	1	5	6
ST45-t037	0	0	1	2	1	1	1	6
ST1357-t030	0	0	0	0	0	5	1	6
ST188-t6367	0	0	0	0	4	1	0	5
ST2139-t189	0	0	0	0	0	2	2	4
ST238-t030	0	0	0	0	3	1	0	4
ST366-t437	0	0	0	0	0	2	2	4
ST5-t037	0	0	1	2	0	0	0	3
ST585-t030	0	0	0	0	0	2	0	2
ST188-t189	0	0	0	0	1	1	0	2
ST1-t114	0	0	0	0	0	2	0	2
ST1057-t002	0	0	0	0	0	1	1	2
Others	3	7	4	26	39	44	19	142

The amount of *S. aureus* isolates identified in each genotype (MLST-spa) in each year were listed.

**Table 4:** Relatedness among SCC*mec*, virulence, and genotypes of clinical *S. aureus*.

		Virulence Associated Genes																	
		*Sea*		*Seb*		*Sec*		*Sed*		*See*		*eta*		*etb*		*tsst*		*pvl*	
SCC*mec*	I/IA	17 */19 #	89.5% ^a^	17/19	89.5% ^a^	12/19	63.2% ^a^	12/19	63.2% ^a^	13/19	68.4% ^a^	12/19	63.2% ^a^	7/19	36.8% ^a^	0/19	0.0% ^b^	5/19	26.3% ^ab^
	II	54/91	59.3% ^c^	28/91	30.8% ^b^	58/91	63.7% ^a^	2/91	2.2% ^b^	21/91	23.1% ^c^	11/88	12.5% ^b^	11/88	12.5% ^b^	26/88	29.5% ^a^	28/88	31.8% ^a^
	III/IIIA	181/237	76.4% ^b^	69/237	29.1% ^b^	77/237	32.5% ^b^	6/237	2.5% ^b^	121/237	51.1% ^b^	39/236	16.5% ^b^	21/236	8.9% ^b^	26/236	11.0% ^b^	49/236	20.8% ^b^
	IV	45/68	66.2% ^bc^	27/68	39.7% ^b^	37/68	54.4% ^a^	2/68	2.9% ^b^	16/68	23.5% ^c^	12/68	17.6% ^b^	5/68	7.4% ^b^	14/68	20.6% ^a^	29/68	42.6% ^a^
	V	11/18	61.1% ^bc^	4/18	22.2% ^b^	5/18	27.8% ^b^	0/18	0.0% ^b^	2/18	11.1% ^cd^	1/17	5.9% ^b^	3/17	17.6% ^b^	0/17	0.0% ^b^	4/17	23.5% ^ab^
	VI	15/31	48.4% ^c^	7/31	22.6% ^b^	10/31	32.3% ^b^	0/31	0.0% ^b^	2/31	6.5% ^d^	3/31	9.7% ^b^	5/31	16.1% ^b^	3/31	9.7% ^b^	9/31	29.0% ^a^
ST types	ST239	222/305	72.8% ^a^	86/305	28.2% ^b^	107/305	35.1% ^a^	4/305	1.3% ^b^	140/305	45.9% ^b^	40/301	13.3% ^b^	35/301	11.6% ^ab^	47/301	15.6% ^b^	69/301	22.9% ^b^
	ST5	20/42	47.6% ^a^	14/42	33.3% ^ab^	20/42	47.6% ^a^	2/42	4.8% ^ab^	10/42	23.8% ^c^	6/40	15.0% ^ab^	1/40	2.5% ^b^	10/40	25.0% ^b^	6/40	15.0% ^b^
	ST45	27/35	77.1% ^a^	13/35	37.1% ^ab^	11/35	31.4% ^a^	1/35	2.9% ^ab^	9/35	25.7% ^c^	5/33	15.2% ^ab^	6/33	18.2% ^a^	3/33	9.1% ^b^	10/33	30.3% ^ab^
	ST59	11/25	44.0% ^a^	11/25	44.0% ^ab^	15/25	60.0% ^a^	3/25	12.0% ^a^	7/25	28.0% ^bc^	4/24	16.7% ^ab^	2/24	8.3% ^ab^	3/24	12.5% ^b^	10/24	41.7% ^a^
	ST546	8/10	80.0% ^a^	3/10	30.0% ^ab^	5/10	50.0% ^a^	0/10	0.0% ^b^	8/10	80.0% ^a^	0/10	0.0% ^b^	2/10	20.0% ^a^	6/10	60.0% ^a^	2/10	20.0% ^b^
	ST366	5/8	62.5% ^a^	0/8	0.0% ^b^	5/8	62.5% ^a^	0/8	0.0% ^b^	0/8	0.0% ^c^	2/8	25.0% ^ab^	0/8	0.0% ^b^	1/8	12.5% ^b^	5/8	62.5% ^a^
	ST1	6/10	60.0% ^a^	6/10	60.0% ^a^	3/10	30.0% ^a^	0/10	0.0% ^b^	1/10	10.0% ^c^	4/10	40.0% ^a^	0/10	0.0% ^b^	0/10	0.0% ^b^	4/10	40.0% ^ab^
	ST188	4/9	44.4% ^a^	6/9	66.7% ^a^	4/9	44.4% ^a^	0/9	0.0% ^b^	1/9	11.1% ^c^	0/9	0.0% ^b^	1/9	11.1% ^ab^	1/9	11.1% ^b^	4/9	44.4% ^a^
*spa* types	t030	81/124	65.3% ^b^	33/124	26.6% ^a^	56/124	45.2% ^ab^	1/124	0.8% ^b^	36/124	29.0% ^b^	23/121	19.0% ^a^	5/121	4.1% ^b^	9/121	7.4% ^b^	29/121	24.0% ^ab^
	t037	131/179	73.2% ^ab^	55/179	30.7% ^a^	55/179	30.7% ^b^	2/179	1.1% ^b^	96/179	53.6% ^a^	17/177	9.6% ^b^	24/177	13.6% ^a^	32/177	18.1% ^a^	38/177	21.5% ^b^
	t437	14/27	51.9% ^b^	8/27	29.6% ^a^	12/27	44.4% ^ab^	1/27	3.7% ^ab^	5/27	18.5% ^ab^	2/27	7.4% ^b^	1/27	3.7% ^b^	8/27	29.6% ^a^	4/27	14.8% ^b^
	t1081	55/68	80.9% ^a^	18/68	26.5% ^a^	23/68	33.8% ^b^	3/68	4.4% ^ab^	30/68	44.1% ^a^	8/65	12.3% ^ab^	14/65	21.5% ^a^	16/65	24.6% ^a^	17/65	26.2% ^ab^
	t002	13/27	48.1% ^b^	9/27	33.3% ^a^	16/27	59.3% ^a^	3/27	11.1% ^a^	6/27	22.2% ^b^	6/27	22.2% ^a^	2/27	7.4% ^ab^	4/27	14.8% ^ab^	11/27	40.7% ^a^

* The numbers represent the amount of strains positive for each virulence associated gene (*sea*, *seb*, *sec*, *sed*, *see*, *eta*, *etb*, *pvl*, and *tsst*) in the first line and the genotypes (SCC*mec*, ST type, *spa* type) at the first column. # The numbers represent the total amount of strains in each SCC*mec* (I, IA, II, III, IIIA, IV, V, VI, and Others), ST (ST239, ST5, ST45, ST59, ST546, ST366, ST1, ST188) or *spa* (t030, t037, t437, t1081,t002) type. The data were adapted to chi-square test and the superscripted “abcd” in the table is the significant difference letter marking method analysis result.

**Table 5:** Primers used in this study.

Primer Name	Sequence (5′-3′)	Target	Amplicon (bp)	Tm (°C)
C1	GATGAGTGCTAAGTGTTAGG	*16S rRNA*	542	55
C2	TCTACGATTACTAGCGATTC			
F1	AAAGCTTGCTGAAGGTTATG	*femA*	823	
F2	TTCTTCTTGTAGACGTTTAC			
M1	GGCATCGTTCCAAAGAATGT	*mecA*	374	
M2	CCATCTTCATGTTGGAGCTTT			
O1	ACCACAATCMACAGTCAT	*orf-X*	212	48
O2	CCCGCATCATTTGATGTG			
ccrB	ATTGCCTTGATAATAGCCITCT	*ccrAB1*	700	48
ccrA1	AACCTATATCATCAATCAGTACGT			
ccrB	ATTGCCTTGATAATAGCCITCT	*ccrAB2*	1000	
ccrA2	TAAAGGCATCAATGCACAAACACT			
ccrB	ATTGCCTTGATAATAGCCITCT	*ccrAB3*	1600	
ccrA3	AGCTCAAAAGCAAGCAATAGAAT			
ccrA4-F	ATGGGATAAGAGAAAAAGCC	*ccrAB4*	1400	
ccrB4-R	TAATTTACCTTCGTTGGCAT			
ccrC-F	ATGAATTCAAAGAGCATGGC	*ccrC*	520	
ccrC-R	GATTTAGAATTGTCGTGATTGC			
mI4	CAAGTGAATTGAAACCGCCT	*mecI-mecR1*	1800	50
mcR3	GTCTCCACGTTAATTCCATT			
IS5	AACGCCACTCATAACATATGGAA	*IS1272-mecA*	2000	52
mA6	TATACCAAACCCGACAAC			
mA2	AACGTTGTAACCACCCCAAGA	*IS431-mecI-mecA*	2000	53
IS2	TGAGGTTATTCAGATATTTCGATGT			
IS431-P4	CAGGTCTCTTCAGATCTACG	*pUB110*	381	55
pUB110 R1	GAGCCATAAACACCAATAGCC			
IS431-P4	CAGGTCTCTTCAGATCTACG	*pT181*	303	52
PT181 R1	GAAGAATGGGGAAAGCTTCAC			
AP1	GGTTGGGTGAGAATTGCACG		Random	38
AP7	GTGGATGCGA			45
ERIC2	AAGTAAGTGACTGGGGTGAGCG			45
arcC-Up	TTGATTCACCAGCGCGTATTGTC	*arcC*	456	55
arcC-Dn	AGGTATCTGCTTCAATCAGCG			
aroE-Up	ATCGGAAATCCTATTTCACATTC	*aroE*	456	55
aroE-Dn	GGTGTTGTATTAATAACGATATC			
glpF-Up	CTAGGAACTGCAATCTTAATCC	*glpF*	465	55
glpF-Dn	TGGTAAAATCGCATGTCCAATTC			
gmk-Up	ATCGTTTTATCGGGACCATC	*gmk*	417	55
gmk-Dn	TCATTAACTACAACGTAATCGTA			
pta-Up	GTTAAAATCGTATTACCTGAAGG	*pta*	474	55
pta-Dn	GACCCTTTTGTTGAAAAGCTTAA			
tpi-Up	TCGTTCATTCTGAACGTCGTGAA	*tpi*	402	55
tpi-Dn	TTTGCACCTTCTAACAATTGTAC			
yqiL-Up	CAGCATACAGGACACCTATTGGC	*yqiL*	516	55
yqiL-Dn	CGTTGAGGAATCGATACTGGAAC			
spa-Up	GTAAAACGACGGCCAGTGCTAAAAAGCTAAACGATGC	*spa*	260	60
spa-Dn	CAGGAAACAGCTATGACCCCACCAAATACAGTTGTACC			

## Data Availability

Not applicable.
